# Supplementary material for: Spanning Fermi arcs in a two-dimensional magnet
Source: Nat Commun. 2022 Sep 9;13:5309. doi: 10.1038/s41467-022-32948-z (PMC9463448; doi:10.1038/s41467-022-32948-z)
Supplement: Supplementary file 1 — Supplementary Information [file 41467_2022_32948_MOESM1_ESM.pdf]

Supplementary Information

Spanning Fermi arcs in a two-dimensional magnet

Ying-Jiun Chen,[1,2,*] Jan-Philipp Hanke,[1,3] Markus Hoffmann,[1,3] Gustav Bihlmayer,[1,3] Yuriy Mokrousov,[1,3,4] Stefan Blügel,[1,3] Claus M. Schneider,[1,2,5] and Christian Tusche[1,2,†]

[1] *Peter Grünberg Institut, Forschungszentrum Jülich, 52425 Jülich, Germany*
[2] *Fakultät für Physik, Universität Duisburg-Essen, 47057 Duisburg, Germany*
[3] *Institute for Advanced Simulation, Forschungszentrum Jülich and JARA, 52425 Jülich, Germany*
[4] *Institute of Physics, Johannes Gutenberg University Mainz, 55099 Mainz, Germany*
[5] *Department of Physics, University of California Davis, Davis, CA 95616, USA*

* yi.chen@fz-juelich.de
† c.tusche@fz-juelich.de

Supplementary Note 1: Photon-energy independent surface Fermi arcs

In order to characterize the dimensionality of the observed Fermi arcs, we carried out momentum microscopy experiments at different photon energies that allows us to probe the various k_z wave vectors perpendicular to the surface. Supplementary Figure 1 shows the photoemission spectral intensity at E_{F} along $k_{\|}, y = 0$ ($\overline{\mathrm{H}} - \overline{\Gamma} - \overline{\mathrm{H}}$) with different photon energies. The lack of the k_z dispersion confirms the 2D nature of the Fermi arcs (denoted by arrows), demonstrating a clear character of surface states. In contrast, highly dispersive bands around $k_{\|}$=0 are evident. This observation agrees well with our first-principles calculations in Fig. 3 of the main text.

Supplementary Note 2: Measured Fermi surface of "bulk" bcc Fe surrounding topological pockets

It was recently predicted that bcc Fe is a topological metal with two disconnected Fermi surface sheets carrying opposite Chern numbers [1, 2]. In order to identify the two non-trivial electron pockets in the Brillouin zone (BZ), measurements for a thick Fe(110) film of twelve MLs were performed. As highlighted in Supplementary Fig. 2, the location and topology of the non-trivial electron pockets are identified at the Fe(110) surface, in a region corresponding to the location of the emergent surface Fermi arcs in 2 MLs Fe/W(110). Note that one would not be able to identify these two non-trivial electron pockets at the Fe(100) surface because they would be overlapping with projected bulk bands [2].

Supplementary Note 3: Orbital texture

We present in Supplementary Fig. 4 the results of our first-principles calculations for the orbital texture around the Fermi arcs that emerge in 2 MLs Fe/W(110) with in-plane magnetization. The distinct topology of these features correlates with the changes of magnitude and direction in the in-plane orbital angular momentum $\mathbf{L}_{\|}$ of the surface arc states as they evolve into the intersection points. This finding is consistent with a recent theoretical [3] and experimental [4] study uncovering that the non-trivial interplay between magnetism and topology imprints locally also on the orbital properties of the electronic states in momentum space.

Supplementary Note 4: Berry curvature

The Berry curvature in momentum space, Eq. (1), is the driving force behind many macroscopic phenomena. Therefore, as shown in Supplementary Fig. 5, we evaluate from first principles the distribution of the Berry curvature $\Omega_{xy}^{\mathbf{kk}}$ around the Fermi arcs on the two opposite sides of the momentum map. Irrespective of a finite overall background, the most important contributions stem from points where the arc surface states evolve into the intersection points (note the pseudo-logarithmic scale in Supplementary Fig. 5). The strong asymmetry in the shape of the Fermi arcs on opposite sides is also present in the distribution of $\Omega_{xy}^{\mathbf{kk}}$.

$$\Omega_{xy}^{\mathbf{kk}}(\mathbf{k}) = -2\,\mathrm{Im}\sum_{n}^{\mathrm{occ}}\sum_{m\neq n}\frac{\left\langle u_{n\mathbf{k}}\middle|\frac{\partial H_{\mathbf{k}}}{\partial k_x}\middle|u_{m\mathbf{k}}\right\rangle\left\langle u_{m\mathbf{k}}\middle|\frac{\partial H_{\mathbf{k}}}{\partial k_y}\middle|u_{n\mathbf{k}}\right\rangle}{(E_{n\mathbf{k}}-E_{m\mathbf{k}})^2}\,. \tag{1}$$

Here, $u_{n\mathbf{k}}$ is an eigenstate of the lattice-periodic Hamiltonian $H_{\mathbf{k}}$ with the energy $E_{n\mathbf{k}}$, and the sum over n is performed over all occupied bands with crystal momentum $\mathbf{k}$.

The formation of the open Fermi arcs in two-dimensional ferromagnets is ascribed to the non-trivial geometry of the mixed phase space of $\mathbf{k}$ and $\widehat{\mathbf{m}}$ in terms of the so-called mixed Berry curvature $\Omega_{ij}^{\widehat{\mathbf{m}}\mathbf{k}}$, Eq. (2), of all occupied states:

$$\Omega_{ij}^{\widehat{\mathbf{m}}\mathbf{k}}(\mathbf{k}) = -2\hat{\mathbf{e}}_{\mathbf{i}}\cdot\,\mathrm{Im}\sum_{n}^{\mathrm{occ}}\left[\widehat{\mathbf{m}}\times\left\langle\frac{\partial u_{\mathbf{k}n}}{\partial\widehat{\mathbf{m}}}\middle|\frac{\partial u_{\mathbf{k}n}}{\partial k_j}\right\rangle\right]. \tag{2}$$

which incorporate derivatives of lattice-period wave functions $u_{\mathbf{k}n}$ with respect to both crystal momentum $\mathbf{k}$ and magnetization $\widehat{\mathbf{m}}$. Here $\hat{\mathbf{e}}_{\mathbf{i}}$ denotes the ith Cartesian unit vector. We used the Wannier interpolation that we

generalized to treat crystal momentum and magnetization direction on an equal footing in order to evaluate the Berry curvature $\Omega^{\mathbf{kk}}$ and $\Omega^{\widehat{\mathbf{m}}\mathbf{k}}$.

Supplementary Note 5: Mixed topology in 2 ML Fe/W(110)

The combination of a complex geometry in real and momentum spaces manifests the non-trivial mixed topology in low-dimensional magnets [3, 5]. The concept of a mixed topology was introduced by formally replacing one of the momentum variable with the magnetization direction, specified by an angle θ in the usual Weyl Hamiltonian. This results in the low-energy description of the system in the composite phase space of entangled crystal momentum $\mathbf{k} = (k_x, k_y)$ and magnetization direction θ by $H = \nu_x k_x \sigma_x + \nu_y k_y \sigma_y + \nu_\theta \theta \sigma_z$, where θ is the angle that the magnetization makes with the z-axis [3, 5].

As demonstrated in Fig. 5 of the main text, to characterize the topological properties in the composite phase space spanned by k_x, k_y, and θ, the momentum Berry curvature $\Omega^{\mathbf{kk}}_{xy}$ and the mixed Berry curvature $\Omega^{\widehat{\mathbf{m}}\mathbf{k}}_{ij}$ are calculated around the Fermi arcs with respect to the magnetization direction, θ. As shown in (a), we find the strong dependence of the Berry curvature on the magnetization direction and in-plane momentum. Remarkably, both Berry curvature $\Omega^{\widehat{\mathbf{m}}\mathbf{k}}_{ij}$ and $\Omega^{\mathbf{kk}}_{xy}$ show the maximum magnitude for $\theta \approx 90°$. Essentially, the sign of the mixed Berry curvature ($\Omega^{\widehat{\mathbf{m}}\mathbf{k}}_{yx}$) changes sharply across the intersection points in the $k_x - k_y$ plane where the surface arcs attach to the projected Weyl nodes. As a result, the Berry curvature field $\boldsymbol{\Omega}$ acquires a monopole-like distribution around the mixed Weyl points. Figure 5b shows the corresponding vector field, where arrows indicate the direction of the curvature field and the colour scale encodes the momentum Berry curvature $\Omega^{\mathbf{kk}}_{xy}$. We find sources of the curvature field at a magnetization angle $\theta \approx 88°$. This characteristic field distribution is related to the presence of topological charges at the mixed Weyl points [5].

Figure 5c shows the band dispersions of valence- and conduction-band states close to the Berry curvature peaks. By applying a detailed evaluation with respect to the magnetization direction θ, we find that two bands only touch at $\theta \approx 88°$, near the Fermi level. This angle is remarkably close to the magnetization direction in the experiment ($\theta = 90°$), pointing approximately along the $-y$ direction. Figure 5d shows the measured spectral function, where the degeneracy points appear at a slightly lower binding energy of 200 meV below the Fermi level. The experimental data further confirms that the observed degeneracy points coincide with the open end of the Fermi arcs in the $k_x - k_y$ momentum space plane of the surface Brillouin zone.

The observation of a monopole-like distribution of the Berry curvature field constitute a strong signature of the topological phase transition and the presence of topological charges around the end points of the open surface arcs in a mixed phase space $(k_x, k_y, \widehat{\mathbf{m}})$. The non-trivial band structure of ultrathin 2D hybrid magnets proves to be a novel arena for exploring new topology physics beyond conventional 3D Weyl semimetals.

Supplementary Note 6: Non-collinear Spin texture of the Fermi arcs

Supplementary Fig. 6 shows the spin resolved intensities for the P_x and P_y spin components along horizontal line profiles across the upper, middle and lower part of the right pair of arcs. A particular pronounced variation of the spin-up and spin-down intensities can be observed for P_x, comparing the sections across the upper and lower part of the Fermi arc. In the left branch of the arc (see black arrows for the arc location) an opposite spin polarization is observed. In the middle section cutting along the $k_y = 0$ symmetry line, the spin polarization vanishes, i.e., the arc has equal spin-up and spin-down intensity contributions. A variation in the right arc branch is less pronounced. This result indicates a significant non-collinearity of the spin texture along the arc and is consistent with our theoretical calculations shown in Fig. 4b of the main text.

The non-collinear spin texture of the Fermi arcs is found to lead to a pronounced variation of the spin direction in the k_x-k_y plane, while no significant variation with the binding energy was observed. Supplementary Figure 7 shows the calculated spin-resolved band structure along on the P_y quantization axis, while the magnetization direction $\mathbf{M}_{-y}$ points anti-parallel to the k_y in-plane direction. The right pair of arcs appears as two co-propagating dark lines, similar as sketched in Fig. 2c of the main text, indicating only a low P_y spin polarization. This is in agreement with the calculated spin texture in Fig. 4b of the main text, as well as the experiment in Fig. 3b of the main text, where only weak blue colours are observed in the right pair of arcs, while a strong spin-down (blue colour) polarization is seen in the left pair of arcs.

The significant non-collinear spin texture along the arcs thus leads to a small P_y component in the right arc close to the $\overline{\mathrm{H}}$-$\overline{\Gamma}$-$\overline{\mathrm{H}}$ line. As a function of the energy, however, characteristic features like the zero crossing of the spin stay unchanged. In particular, this result shows that the spin texture along the Fermi arcs mainly varies as a function of k_x and k_y, as displayed in the spin polarization maps in Figs. 3b and 4 of the main text.

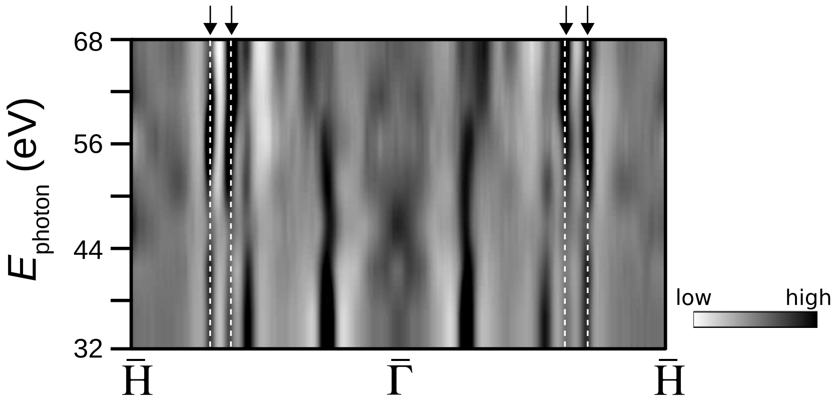

Supplementary Fig. 1. Photon-energy (k_z) independent surface arc states. Photoemission spectral intensity map at E_F along $k_\parallel, y = 0$ ($\overline{\mathrm{H}} - \overline{\Gamma} - \overline{\mathrm{H}}$) for s-polarized light with a photon energy range of 32-68 eV that probes the various k_z wave vectors perpendicular to the surface. The observed negligible k_z dispersion of Fermi arcs denoted by the arrows confirms their 2D nature, showing a clear signature of surface states. White dashed lines serve as a guide to the eye.

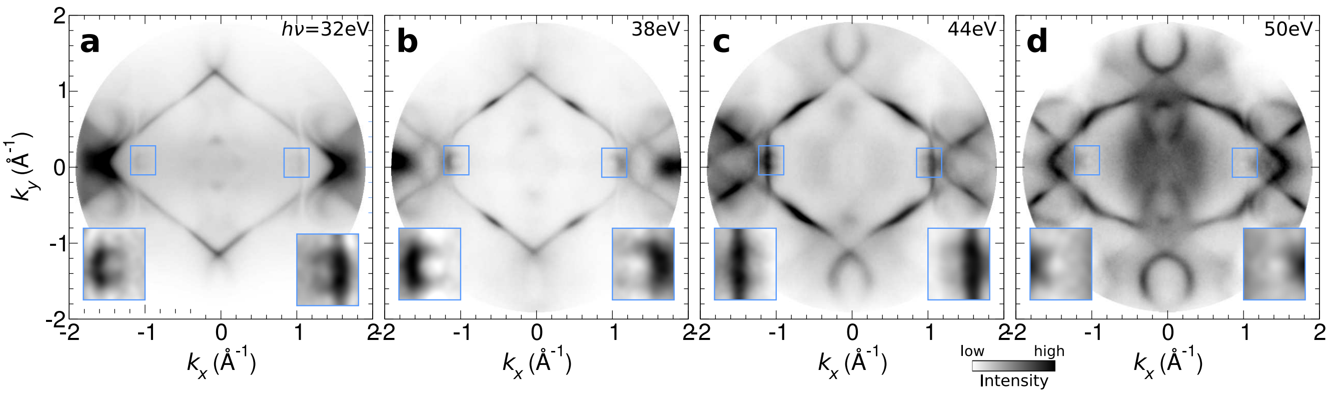

Supplementary Fig. 2. Topological electron pockets in "bulk" bcc Fe. Measured 2D photoemission momentum map at the Fermi energy E_F for a 12 MLs Fe(110) film measured using s-polarized light with a photon energy of **(a)** 32 eV, **(b)** 38 eV, **(c)** 44 eV and **(d)** 50 eV, surrounding the two topological electronic pockets denoted by the blue rectangles. The inset shows the electron pockets at higher magnification. The intensity is displayed on a linear grey scale.

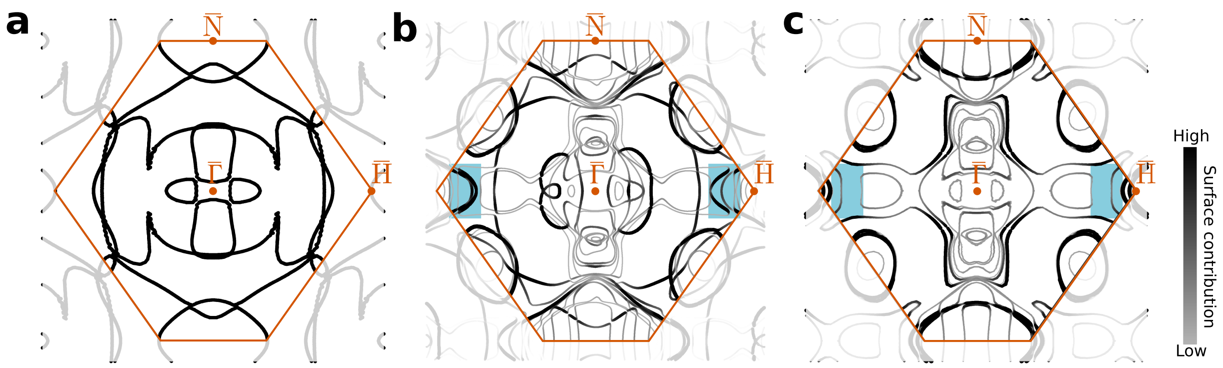

Supplementary Fig. 3. Theoretical Fermi surface of free-standing Fe(110) monolayers. Theoretical Fermi surface of (**a**) a free-standing 2ML Fe(110), (**b**) 2ML Fe/W(110), and (**c**) bulk W(110). The shaded blue regions highlight the emergent Fermi arc surface states in 2 ML Fe/W(110), which is located within the band gap of bulk W(110) and is absent in a free-standing film.

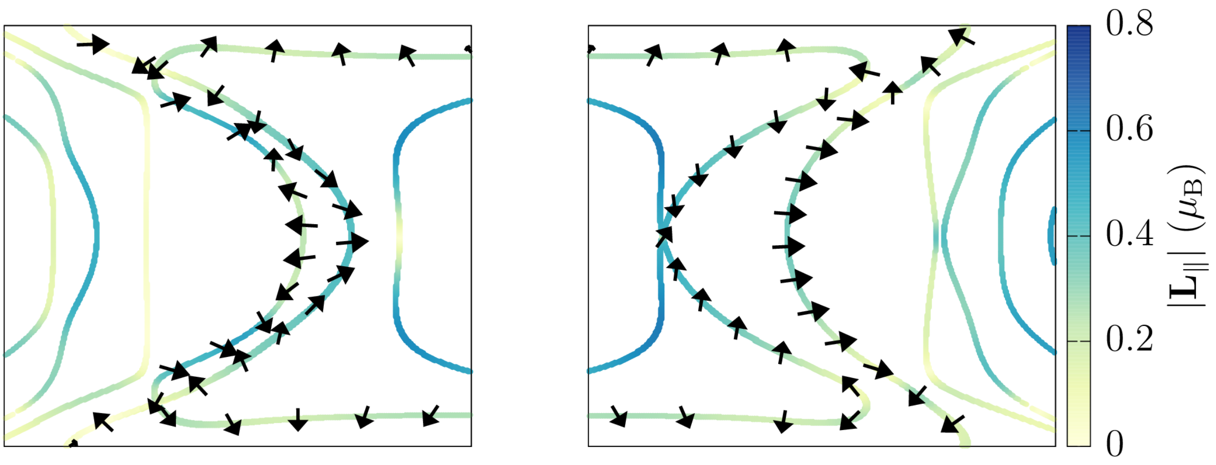

Supplementary Fig. 4. Orbital texture around the Fermi arcs in 2 MLs Fe/W(110). The colour scale represents the magnitude of the local in-plane orbital moment $\mathbf{L}_{\parallel}$ in units of Bohr magneton, and unit vectors indicate its direction in the regions of the Fermi arcs on opposite sides of the momentum map.

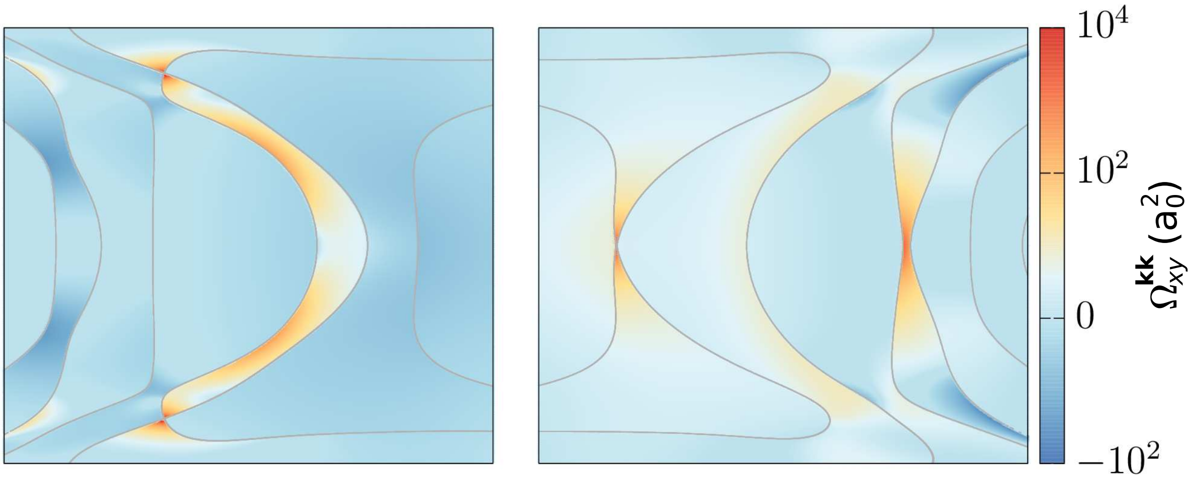

Supplementary Fig. 5. Berry curvature around the Fermi arcs in 2 MLs Fe/W(110). a, The local geometrical curvature $\Omega^{\mathbf{kk}}_{xy}$ in the regions of the Fermi arcs is indicated by a pseudo-logarithmic colour scale. Grey lines represent the Fermi surface contour on the two sides of the momentum map.

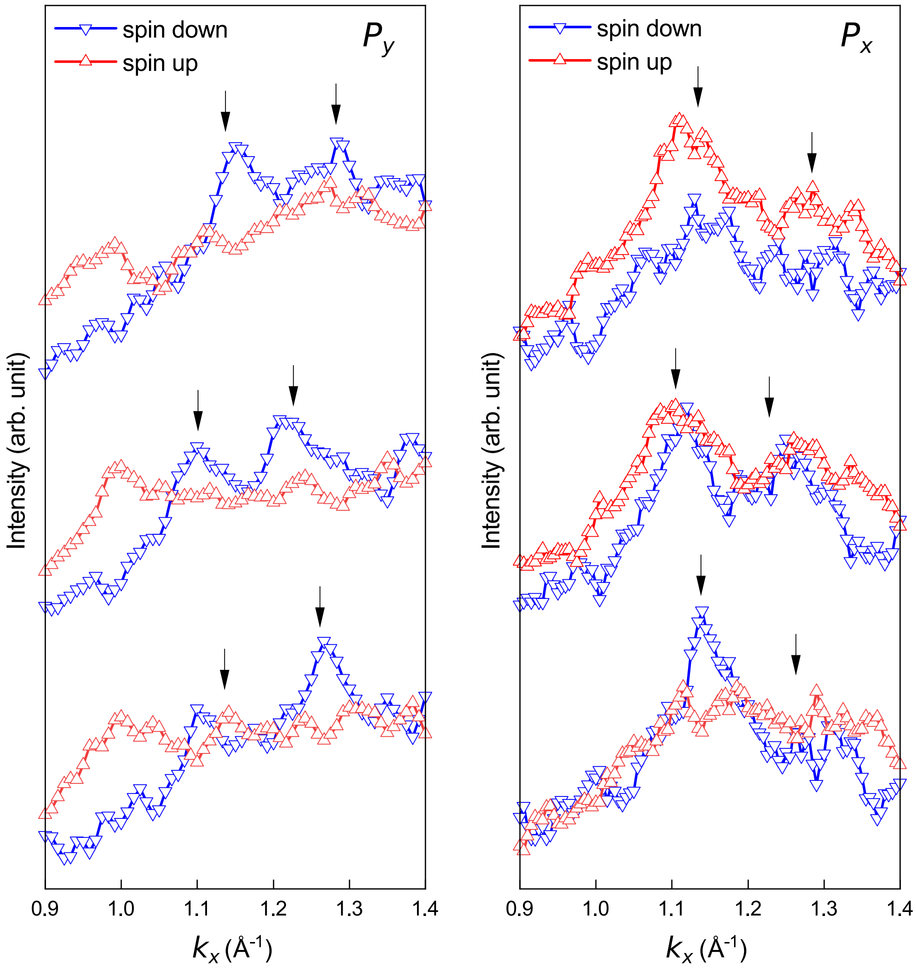

Supplementary Fig. 6. Spin resolved intensity profiles across the right pair of arcs. Horizontal intensity profiles across the upper ($k_y = +0.18$Å^{-1}), middle ($k_y = 0$) and lower ($k_y = -0.18$ Å^{-1}) part of the right pair of arcs. The sample magnetization direction M_{-y} points anti-parallel to the k_y axis. Red and blue curves denote spin-up and spin-down intensities, respectively, with respect to the vertical (P_y, left panel) and horizontal (P_x, right panel) spin quantization axis.

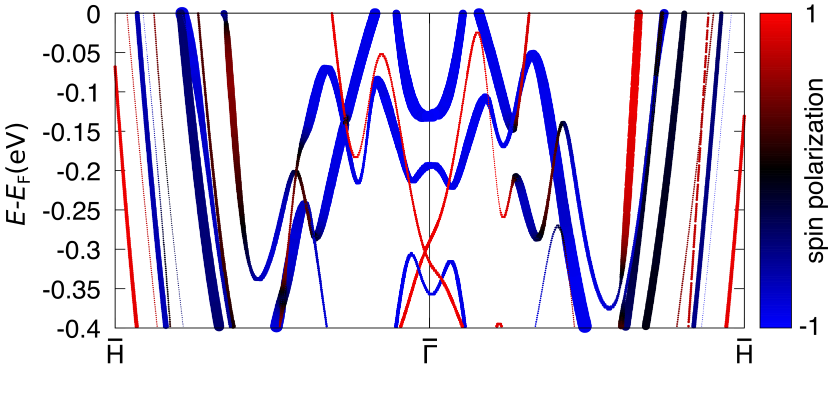

Supplementary Fig. 7. Theoretical spin resolved band dispersion. Calculated band dispersion of 2ML Fe/W(110) plotted along the $\overline{\mathrm{H}}$-$\overline{\Gamma}$-$\overline{\mathrm{H}}$ direction in the surface Brillouin zone. Red and blue colours denote spin-up and spin-down states with respect to the P_y spin quantization axis, respectively. The sample magnetization $\mathbf{M}_{-y}$ points anti-parallel to the k_y direction. The right pair of arcs appears as two co-propagating dark lines, indicating only a low P_y spin polarization.

[1] Gosálbez-Martínez, D., Souza, I. & Vanderbilt, D. Chiral degeneracies and Fermi-surface Chern numbers in bcc Fe. *Phys. Rev. B* **92**, 085138 (2015).

[2] Gosálbez-Martínez, D., Autès, G. & Yazyev, O. V. Topological fermi-arc surface resonances in bcc iron. *Phys. Rev. B* **102**, 035419 (2020).

[3] Niu, C. *et al.* Mixed topological semimetals driven by orbital complexity in two-dimensional ferromagnets. *Nat. Commun.* **10**, 3179– (2019).

[4] Min, C.-H. *et al.* Orbital fingerprint of topological Fermi arcs in the Weyl semimetal TaP. *Phys. Rev. Lett.* **122**, 116402 (2019).

[5] Hanke, J.-P., Freimuth, F., Niu, C., Blügel, S. & Mokrousov, Y. Mixed Weyl semimetals and low-dissipation magnetization control in insulators by spin-orbit torques. *Nat. Commun.* **8**, 1479– (2017).
